# Heterologous Expression of PKPI and Pin1 Proteinase Inhibitors Enhances Plant Fitness and Broad-Spectrum Resistance to Biotic Threats

**DOI:** 10.3389/fpls.2020.00461

**Published:** 2020-04-30

**Authors:** David Turrà, Stefania Vitale, Roberta Marra, Sheridan L. Woo, Matteo Lorito

**Affiliations:** ^1^Department of Agricultural Sciences, University of Naples Federico II, Naples, Italy; ^2^Task Force on Microbiome Studies, University of Naples Federico II, Naples, Italy; ^3^Institute for Sustainable Plant Protection, National Research Council, Naples, Italy; ^4^Department of Pharmacy, University of Naples Federico II, Naples, Italy

**Keywords:** *Pin1*, *PKPI*, plant cell proliferation, *Pseudomonas syringae*, *Alternaria alternata*, *Botrytis cinerea*, disease resistance

## Abstract

Kunitz-type (PKPI) and Potato type I (Pin1) protease inhibitors (PIs) are two families of serine proteinase inhibitors often associated to plant storage organs and with well known insecticidal and nematicidal activities. Noteworthy, their ability to limit fungal and bacterial pathogenesis *in vivo* or to influence plant physiology has not been investigated in detail. To this aim, we generated a set of PVX-based viral constructs to transiently and heterologously express two potato *PKPI* (*PKI1*, *PKI2*) and three potato *Pin1* (*PPI3A2*, *PPI3B2*, *PPI2C4*) genes in *Nicotiana benthamiana* plants, a widely used model for plant-pathogen interaction studies. Interestingly, transgenic plants expressing most of the tested PIs showed to be highly resistant against two economically important necrotrophic fungal pathogens, *Botrytis cinerea* and *Alternaria alternata*. Unexpectedly, overexpression of the *PKI2* Kunitz-type or of the *PPI2C4* and *PPI3A2* Potato type I inhibitor genes also lead to a dramatic reduction in the propagation and symptom development produced by the bacterial pathogen *Pseudomonas syringae*. We further found that localized expression of *PPI2C4* and *PKI2* in *N. benthamiana* leaves caused an increase in cell expansion and proliferation which lead to tissue hypertrophy and trichome accumulation. In line with this, the systemic expression of these proteins resulted in plants with enhanced shoot and root biomass. Collectively, our results indicate that PKPI and Pin1 PIs might represent valuable tools to simultaneously increase plant fitness and broad-spectrum resistance toward phytopathogens.

## Introduction

PKPI and Pin1 are among the most abundant naturally occurring plant serine proteinase inhibitors (PIs). Large amounts of these inhibitors accumulate in plant reproductive and storage organs, as in the case of *Solanum tuberosum* (Heibges et al., 2003; van den Broek et al., 2004). Nevertheless, their genes are also transcribed, however at lower levels, in all other plant tissues (Kuo et al., 1984; Lincoln et al., 1987; Jofuku and Goldberg, 1989; Heitz et al., 1993; Wang et al., 2003, 2008; Turrà et al., 2009). Serine PIs expression apart from being regulated at developmental, spatial and species-specific level (Lee et al., 1986; Rosahl et al., 1986; Balandin et al., 1995; Singh et al., 2009; Tamhane et al., 2009; Turrà et al., 2009), is boosted up by various external stimuli including wounding, insect feeding and microbial infections (Mello et al., 2001; van Loon et al., 2006; Turrà and Lorito, 2011), being one of the best-characterized defense reactions activated by the plant in response to pathogen and insect attack. Many studies on the effect of serine PIs, either artificially introduced into diets or heterologously expressed in transgenic plants, have shown the ability of these proteins to reduce the growth and development of a wide range of herbivorous insects and pathogenic nematodes mainly by interfering with nutrient digestibility and fertility (Jongsma et al., 1995; Urwin et al., 1995; Altpeter et al., 1999; Andrade et al., 2003; Cai et al., 2003; Srinivasan et al., 2005).

Besides, few serine PIs have also shown inhibitory activity against bacteria and fungi *in vitro* by reducing their growth or conidial germination and hyphal swelling, respectively (Lorito et al., 1994; Dunaevsky et al., 1996; Chen et al., 1999; Soares-Costa et al., 2002; Kim et al., 2005; Hermosa et al., 2006; Kim et al., 2006; Di Cera, 2009). However, while serine PIs insecticidal and nematicidal activities have been efficiently proven *in planta*, their ability to alter plant resistance against fungal or bacterial pathogens *in vivo* has remained elusive.

Proteases and PIs play important roles in plant-pathogen interactions; nevertheless, testimony for their endogenous role in plants is relatively recent and our current understanding of the diverse physiological processes regulated by PIs is rapidly expanding (van der Hoorn, 2008; Turrà and Lorito, 2011; Grosse-Holz and van der Hoorn, 2016). To date, protease-PI interactions have been shown to regulate many diverse aspects of the plant life cycle including senescence and programmed cell death (PCD), leaf trichome density and branching, seed and flower development and sieve element maturation (Solomon et al., 1999; Xu et al., 2001; Sin and Chye, 2004; Pak and Van Doorn, 2005; Liu et al., 2006; Xie et al., 2007; Luo et al., 2009; Boex-Fontvieille et al., 2015; Rustgi et al., 2017).

As proteolysis is a fundamental process in all living beings, in order to avoid undesired side-effects plants must carefully control endogenous protease activity in both a timely and a spatial manner (van der Hoorn, 2008; Turrà and Lorito, 2011). In a previous work from our group, we have shown that *PI* gene members of the *Pin1* and *PKPI* families are differentially expressed in *Solanum tuberosum* var. Desireè plants upon abiotic or biotic insults, or in a tissue-dependent manner, thus indicating a possible role for these PIs as both endogenous- and defense-related plant regulators (Turrà et al., 2009).

In this study, the effect of transient expression of different members of the *Pin1* and *PKPI* families on plant resistance toward fungal and bacterial pathogens and on plant physiology is reported. When heterologously expressed in *Nicotiana benthamiana*, different potato *PKPI* and *Pin1* genes confer protection against *B. cinerea* and *A. alternata*, two agronomically important pathogens. Moreover, *in vivo* assays designed to challenge PIs-expressing plants with *Pseudomonas syringae* pv. *tabaci* also revealed enhanced plant resistance to bacterial attack. In addition to this, overexpression of two of these serine *PI* genes also caused severe developmental effects on *N. benthamiana* plants, including over-accumulation of trichomes and growth enhancement. These phenotypes were accompanied by the high inhibitory activity of total soluble proteins (TSP) extracted from transformed leaf patches toward yet unknown proteases present in the *N. benthamiana* leaf apoplast. Based on these results, we propose that Pin1 and PKPIs are critically involved in host resistance and modulation of plant physiology.

## Materials and Methods

### Microbial Strains, Plants, and Culture Conditions

*Nicotiana benthamiana* plants were cultivated and maintained at 25°C in a phytocabinet under 16/8 h light-dark photoperiod.

*Agrobacterium tumefaciens* GV3101, *Escherichia coli* DH5α and a rifampicin-resistant strain of *P. syringae* pv. *tabaci* were routinely grown in Luria-Bertani (LB) media (Sambrook and Russell, 2001) with appropriate antibiotics at 28°C, 37°C, or 28°C, respectively. All bacterial DNA transformations were performed by electroporation using standard protocols (Sambrook and Russell, 2001).

Conidia of the pathogenic fungi *B. cinerea* and *A. alternata* were harvested respectively from malt extract peptone agar (MEP) (Difco, Detroit, MI, United States) or potato dextrose agar (PDA) (Sigma-Aldrich, St. Louis, MO, United States) plates, after 1 week of incubation at 25°C, as previously described (Hermosa et al., 2006).

### Construction of PVX::*PI* Gene Fusions

To amplify full-length cDNAs of *PKI1*, *PKI2*, *PPI3A2*, *PPI3B2*, and *PPI2C4* genes (Hermosa et al., 2006; Turrà et al., 2009), total RNA was extracted from 100 mg of *Solanum tuberosum* var. Desireè sprouts using the TRI Reagent (Ambion, Austin, TX, United States). First-strand cDNA was synthesized using the Reverse Transcription System kit (Promega, Madison, WI, United States) and 1 μg random primers for every 2 μg of total RNA, following the supplier’s instructions. The *PKPI* and *Pin1* derivatives were amplified by PCR using the oligonucleotide combinations indicated in Supplementary Table S1, subcloned into pGEM-T Easy vector (Promega), and ligated into the *Cla*I and *Sal*I sites of the *A. tumefaciens* binary PVX vector pGR106 (Lu et al., 2003).

Constructs containing the inserts in sense orientation were designated PVX::*PKI1*, PVX::*PKI2*, PVX::*PPI3A2*, PVX::*PPI3B2*, and PVX::*PPI2C4*. The obtained binary vectors, the pGR106 vector without any insert and the pGR208 vector (Rairdan et al., 2008), *gfp* cDNA ligated in the same vector, were transformed into *A. tumefaciens* strain GV3101. pGR106 and pGR208 vectors were used as PVX controls.

### Transient Expression of *PI*s Genes in *N. benthamiana* and *in vivo* Resistance Assay on PVX-Infected Plants

*N. benthamiana* seedlings at the fourth true-leaf stage were used for *A. tumefaciens* infiltration. To evaluate local effects of PI overexpression, *A. tumefaciens* overnight cultures diluted to an OD600 of 0.25 with sterile distilled water were used to infiltrate the abaxial side of the leaf (using a needleless 5 ml syringe). Alternatively, to achieve systemic transformation of plants, third and fourth leaves of 2–3-week-old *N. benthamiana* seedlings were wounded twice along the midvein with a sterile wooden toothpick previously streaked over an *A. tumefaciens* culture grown on solid agar medium.

For *P. syringae* infection, 5 days after *A. tumefaciens* syringe-infiltration, the same leaf areas were infiltrated (the abaxial side of the leaf) with 40 μl of a 1 × 10^8^ *P. syringae* cells/ml culture (adjusted with sterile distilled H_2_O to OD600 = 0.24). After 2, 4, 7, and 9 days, the necrotic zone around the inoculation site was imaged. Every treatment was repeated at least 6 times and on at least three different plants. A separate round of experiments was used to quantify *P. syringae* growth in the agroinfiltrated leaves. Ten μl of a *P. syringae* cell suspension (OD600 = 0.24) was applied to needle-pricked leaves. Plants were covered with clear polyethylene bags and sealed around the base using elastic bands, to keep humidity. After 1, 3, and 6 days, six leaf discs (0.8 cm diameter) per treatment were excised from inoculated areas, pooled and ground (Ultra-Turrax T25 basic, IKA Labortechnik, Germany) in 10 mM MgSO_4_ (1 ml/disc), by keeping the tube in an ice bath. An aliquot of the homogenate was plated on LB-rifampicin agar at three different dilutions (10^–3^, 10^–4^, and 10^–5^) and colonies were counted 1 and 2 days after incubation at 28°C. Another aliquot (15 μl) of the homogenate was mixed with 110 μl of LB-rifampicin and incubated in 96-well microtiter plates at 28°C (120 rpm for 16 h). Optical density at 550 nm was then measured with a Bio-Rad microplate reader (Bio-Rad, Richmond, CA, United States). *P. syringae* inoculation was independently repeated on at least six leaves of at least three different plants.

For *B. cinerea* and *A. alternata* infections, upper leaves of systemically transformed *N. benthamiana* plants (11 days after *A. tumefaciens* toothpick inoculation) were challenged with 10 μl of germination solution (20 mM glucose, 20 mM potassium phosphate) containing 10^5^ or 10^7^ conidia/ml of *B. cinerea* or *A. alternata*, respectively. All plants were covered with transparent polyethylene bags and sealed around the base using elastic bands, to keep humidity. The appearance of necrotic spots was assessed 2, 4, and 6 days after inoculation and disease incidence recorded.

Each pathogen–PVX construct combination was assayed on at least four different leaves of at least three different plants. All infection assays were repeated at least twice. Data are presented as mean values ± SD of different experiments. To assess statistical differences between control (PVX::*gfp*) and PI expressing samples a Yates’ corrected chi-squared test (two-sided) was used. Statistical differences between treatments at each time point were assessed by one-way ANOVA with *post hoc* Tukey HSD Tests.

### RT-PCR Analysis of PIs Expression

For RT-PCR validation of transient PI expression, total RNA was isolated from 100 mg of control (PVX::*gfp*) and PIs transformed *N. benthamiana* leaves (5 days post agroinfiltration) using the TRI Reagent (Ambion). RT-PCR was performed on equal amounts of total RNA using the Reverse Transcription System kit (Promega) and of 1 μg random primers for each 2 μg total RNA, following the supplier’s instructions. Two microliters of first-strand cDNA solution were used as a template for RT-PCR experiments. Amplifications of *Pin1*, *PKPI*, and β*-tubulin* gene transcripts were performed as indicated earlier (Turrà et al., 2009). Plasmid DNA of the cloned cDNAs *PPI3B2* and *PKI1* (Turrà et al., 2009) were used as positive controls and PCR products of the constitutively expressed β*-tubulin* gene used as a quantitative control. Amplifications were repeated in independent occasions on neo-synthesized cDNA from at least three independently repeated experiments.

### Protein Extractions

For the extraction of the TSP, ten grams of untransformed or of locally transformed *N. benthamiana* leaf areas (7 days after *A. tumefaciens* syringe-inoculation) were ground with an Ultra-Turrax Homogenizer in 30 ml of 0.1 M sodium phosphate buffer (pH 6.8) by keeping the tube in an ice bath. The slurry was incubated 1h on ice under occasional shaking, filtered through four layers of cheesecloth, and cleared by centrifugation at 50,000 g for 1 h at 4°C in a Beckman L7-65 Ultracentrifuge (Beckman, Milan, Italy).

Apoplastic fluids (AF) were prepared from *N. benthamiana* leaves according to the method of Moehnke et al. (2008), with minor modifications. Briefly, 10 g of leaf material was vacuum-infiltrated for 2 min with 100 ml infiltration buffer [100 mM Tris/HCl (pH 7.5), 10 mM MgCl_2_]. Leaves were then dried with sterile paper towels and placed into the barrel of a 50 ml syringe. The syringe was subsequently inserted into a 50 ml falcon tube with the needle hub facing downwards and spun at 2,000 g for 10 min at 4°C. After centrifugation, AF was collected from the bottom of the centrifuge tube.

All protein extracts were subjected to filtration and dialysis by using Centriprep YM-3 devices (Amicon Corporation, Danvers, MA, United States), filter sterilized (0.22 μm) and stored at −20°C if not immediately used. Protein concentrations were determined by a Bradford DC protein assay (Bio-Rad) using bovine serum albumin as a standard.

### *In vitro* Evaluation of Plant Crude Extracts Activity

Inhibition assays of *N. benthamiana* TSP and AF proteolytic activities by TSP extracted from PVX::*gfp*, PVX::*PKI2*, and PVX::*PPI2C4* transformed *N. benthamiana* leaf areas were carried out in microtiter plates by using azocasein (Sigma-Aldrich) as chromogenic substrate and in-gel protease assays using the Bio-Rad zymogram buffer system, following the previously described procedures (Tian et al., 2004; Hermosa et al., 2006). For the first method, a total of 20 μg of TSP and AF from untransformed plants were preincubated with 20 μg of TSP from PVX-transformed plants in a volume of 250 μl for 30 min at room temperature, and followed by incubation with 200 μl of 1% azocasein (w/v) at 37°C for 1 h. The reaction was halted by adding an equal volume of 10% (w/v) TCA. After 10 min on ice, the reaction mixture was centrifuged for 10 min at 13,000 g and the supernatant mixed with an equal volume of 1 M NaOH. The optical density at 450 nm was then determined with a Bio-Rad microplate reader. The percentage of the remaining protease activity was therefore plotted relative to that of TSP and AF samples from untransformed plants incubated with TSP extracted from PVX:*gfp* transformed plants. Experiments were performed in triplicate and repeated in at least two independent occasions. Data represent the mean value [±SD (standard deviation)] across all experiments. Statistical differences between treatments were assessed by one-way ANOVA with *post hoc* Tukey HSD Tests.

For zymogen in-gel protease assays, 20 μg of TSP from mock- (H_2_O) and PVX-infiltrated *N. benthamiana* leaf areas were mixed with zymogram sample buffer and loaded on a 10% SDS-polyacrylamide gel without boiling or addition of reducing reagents. Following electrophoresis, the gel was incubated in 1x zymogram renaturation buffer for 30 min. Then the gel was incubated in 1x zymogram development buffer for 18 h at 37°C before staining with 0.5% Coomassie Brilliant Blue. Areas of protease activity were revealed as cleared bands on a blue background.

To assess the antifungal activity of plant crude extracts, 10 μl of a solution of 10^7^ conidia/ml of *B. cinerea* or *A. alternata* were mixed with 40 μl of plant crude extracts (50 μg/ml) and 40 μl of Potato Dextrose Broth (PDB). After 48 h of incubation at 28°C in a 96-well microtiter plate, the change in optical density at 550 nm was determined using a Bio-Rad microplate reader. Each experiment was repeated at least three times and data presented as the percentage of growth inhibition relative to that of the PVX:*gfp* transformed plants. Data correspond to mean values (± SD) across all experiments. Statistical differences between treatments were assessed by one-way ANOVA with *post hoc* Tukey HSD Tests.

### Determination of Plant Growth and Analysis of Leaf Surface Expansion

The effect of PI transient expression on *N. benthamiana* shoot and root growth was evaluated in pot experiments. Seedlings were grown *in vitro* at 23°C under 24 h fluorescent lighting (3,500–6,000 lux) on Murashige and Skoog (MS) salt medium (ICN Pharmaceuticals Inc., Cleveland, OH, United States), and 1% bacto-agar (Difco, Detroit, MI, United States) first and then transferred to soil in 10 cm diameter pots and left to grow in a phytocabinet as described above. Plantlets at the fourth-leaf stage were *A. tumefaciens* toothpick inoculated, as described above. Complete shoots and roots were collected separately (21 days after agroinfection). Roots were briefly rinsed to remove attached sand, quickly dried with a paper towel, incubated for 72 h at 75°C and weighted to estimate the dry weight. Experiments were repeated twice (*n* = 10). Data correspond to mean values (± SD) across all experiments. Statistical differences between treatments were assessed by one-way ANOVA with *post hoc* Tukey HSD Tests.

To determine leaf disc surface expansion and leaf strip curvature of PI transformed leaf areas, the methods were adapted from those of Gevaudant and coworkers (Gevaudant et al., 2007). Briefly, fully developed PVX-transformed *N. benthamiana* leaf areas (7 days after *A. tumefaciens* syringe-inoculation) were used. Leaf discs (1 cm diameter) and strips (2 × 10 mm) were cut from the interveinal region and incubated for 24 h at room temperature in 10 mM Sucrose, 10 mM KCl, and 0.5 mM 2-(N-Morpholino)ethanesulfonic acid hemisodium salt (MES), pH 6.0. Leaf discs were photographed with a digital camera before and after the treatment and their surface estimated as their pixel content, by the use of the ImageJ software (Collins, 2007). Differences in surface increase between treatments and controls (PVX::*gfp*) were expressed as a percentage of the initial disc area. Leaf strip curvature was estimated as the angle made by the two tangents to the two terminal parts of each strip. Each experiment was repeated three times on at least 15 leaf discs or strips per treatment. Statistical differences between treatments were assessed by one-way ANOVA with *post hoc* Tukey HSD Tests.

For microscopic analysis of cell size and nuclei density, adaxial epidermis from agroinfiltrated (7, 13, and 20 days after *A. tumefaciens* syringe-infiltration) *N. benthamiana* leaf areas was used. Briefly, entire leaves were detached and immersed for 1 h in 1% (v/v) Tween 20 before peeling off the adaxial epidermis from the agro-infiltrated area. Tissues were mounted in water and observed with an Axioskop2 Plus microscope (Zeiss, Milan, Italy). Cell size measurement was performed by using the ImageJ software (Collins, 2007). For nuclear visualization, leaf epidermis was stained with 4′,6-diamidino-2-phenylindole (DAPI; 1 μg/ml) for 20 min, and mounted in 50% (v/v) phosphate-buffered saline (PBS)-glycerol for observation. Quantification of epidermial cell size and nuclear density was repeated at least four times on 500 cells/leaf per treatment. To assess statistical differences between control (PVX and PVX:*gfp*) and PI expressing samples a Yates’ corrected chi-squared test (two-sided) was used.

### Sequence Data and Bioinformatic Analysis

BLAST searches were performed in the NCBI database^1^. Signal peptide and extracellular localization predictions were performed by using the SignalP and WoLF PSORT softwares, respectively (Emanuelsson et al., 2007; Horton et al., 2007).

Sequence data from this article can be found in the GenBank data library under accession numbers DQ087220 (*PKI1*), JX878493 (*PKI2*), DQ087224 (*PPI3A2*), DQ087221 (*PPI3B2*), DQ087223 (*PPI2C4*).

## Results

### Potato *Kunitz* and *Pin1* PIs Are Efficiently Expressed in *Nicotiana benthamiana* Plants

Five PCR fragments corresponding to three previously characterized (*PPI3A2*, *PPI3B2*, and *PPI2C4*) full-length *Pin1* cDNAs and two full-length *Kunitz* cDNAs, one of them previously characterized (*PKI1*) and a novel one (*PKI2*) were amplified from potato sprouts (Turrà et al., 2009). BlastX analysis of the latter gene indicated about 95% identity with the *S. tuberosum P1H5* gene (AAM10743) and 94% identity with *PKI1*. All amplified PIs were predicted to be secreted to the apoplast according to the SignalP and WoLF PSORT prediction tools.

**FIGURE 1:**
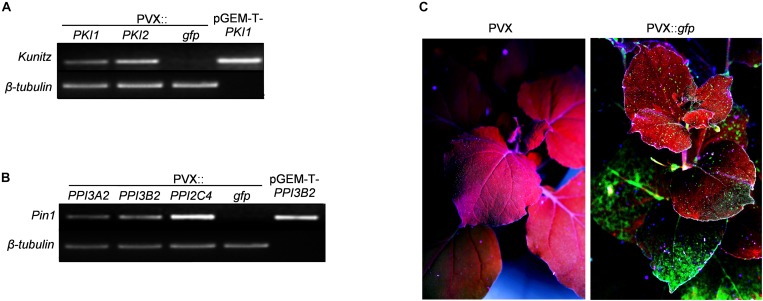
Functional validation of PVX-mediated gene expression. **(A,B)** RT-PCR analysis of potato *PKPI* and *Pin1* expression in control (PVX::*gfp*) and in *PKI1*, *PKI2* **(A)** or *PPI3A2*, *PPI3B2*, *PPI2C4* **(B)** transiently expressing *Nicotiana benthamiana* plants. Total RNA was extracted from *N. benthamiana* leaves 5 days after *Agrobacterium tumefaciens* infiltration and subjected to RT-PCR analysis. Plasmids pGEM-T-*PKI1* **(A)** and pGEM-T*-PPI3B2* **(B)** were used as positive amplification controls. The *β-tubulin* gene was used to confirm an equal amount of total RNA among samples. Each sample consisted of leaf tissues pooled from at least four independently transformed plants. Representative results from three independent experiments are shown. **(C)** Systemic transgene expression was validated by analyzing GFP fluorescence in newly formed *N. benthamiana* leaves at different time intervals 11 days after toothpick inoculation of basal leaves with a colony of *A. tumefaciens* carrying either the pGR106 (PVX control) or the pGR106-*gfp* (PVX::*gfp*) plasmid. Photographs in **(C)** are representatives of UV-irradiated *N. benthamiana* plants 11 dpi.

Recombinant PVX plasmids containing the full-length cDNA of all of the above mentioned *PI* genes were used for transient transformation of *N. benthamiana* plants. For local expression of *PI* genes, the method of transient *Agrobacterium*-mediated expression by leaf infiltration was chosen. Transgene expression in agroinfiltrated leaf patches was confirmed 5 days after agro-infiltration by extracting total RNA and performing RT-PCR using PI specific oligonucleotides (Supplementary Table S1) followed by DNA sequence analysis (Figures 1A,B). For systemic transgene expression, a toothpick-inoculation system was used. In this case transgene expression in the upper non-inoculated parts of the plant was verified by monitoring GFP fluorescence 5, 7, 11, 14, and 21 DPI with the PVX::*gfp* viral construct. All inoculated plants displayed systemic GFP expression starting from 11 DPI (Figure 1C).

### Transient Expression of *Kunitz* and *Pin1* Inhibitor Genes Increases Plant Resistance Toward the Fungal Pathogens *Botrytis cinerea* and *Alternaria alternata*

**FIGURE 2:**
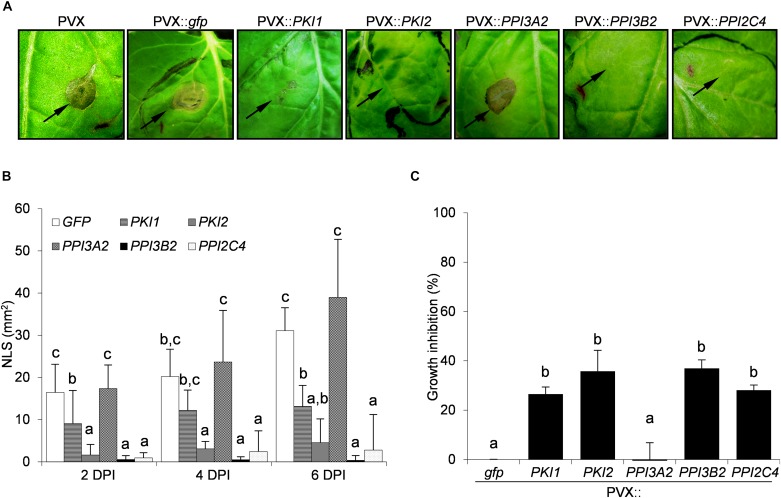
Transient expression of *PKI2* Kunitz and of *PPI3B2* and *PPI2C4* Pin1 inhibitor genes increases plant resistance toward the fungal pathogen *Botrytis cinerea*. Third and fourth leaves of *Nicotiana benthamiana* plants were toothpick inoculated with *Agrobacterium tumefaciens* carrying the indicated viral constructs. After 11 days, the upper leaves showing systemic viral symptoms were challenged with 10 μl of germination solution (20 mM glucose, 20 mM potassium phosphate) supplemented with 10^5^ conidia/ml of *Botrytis cinerea*. **(A)** Representative pictures of necrotic lesions (NLS) observed 6 days after *B. cinerea* inoculation on the indicated PVX-transformed *N. benthamiana* plants. **(B)** Severity of necrotic lesions recorded 2, 4, and 6 days after *B. cinerea* inoculation (DPI) on the indicated PVX-transformed *N. benthamiana* plants. Values are the means of four independent inoculations repeated on at least three plants; error bars represent the standard deviation. Mock inoculated plants (H_2_O), PVX and PVX::*gfp* transformed plants were used as negative controls. Different letters indicate significant differences (*P* < 0.01) among treatments at each DPI according to One-way ANOVA with *post hoc* Tukey HSD Test. **(C)** *In vitro* inhibitory activity of total soluble proteins (TSP; 50 μg/ml) extracted from PVX-transformed *N. benthamiana* leaf areas toward *B. cinerea*. Fungal growth inhibition was measured as the reduction of the optical density at 550 nm relative to the control treatment (TSP extracted from PVX::*gfp* transformed plants). Values are the means of triplicate determinations; error bars represent the standard deviation. Different letters indicate significant differences (*P* < 0.05) among the different treatments according to One-way ANOVA with *post-hoc* Tukey HSD Test.

**FIGURE 3:**
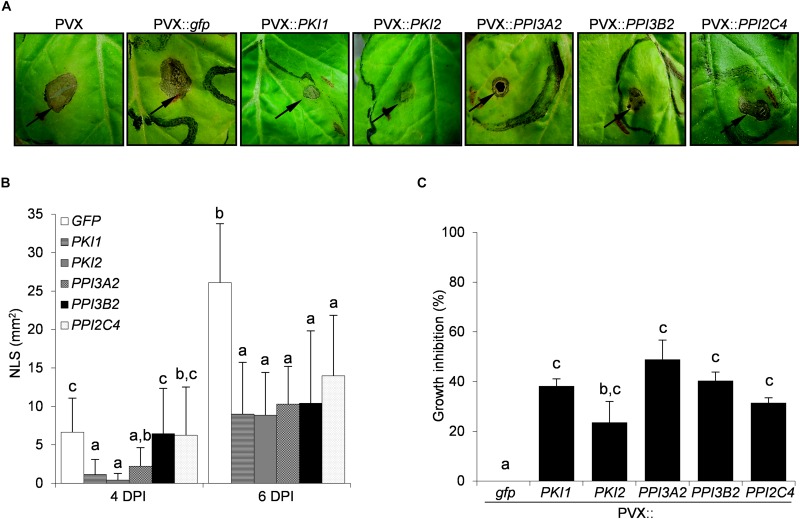
Transient expression of several *Kunitz* and *Pin1* genes increases plant resistance toward the fungal pathogen *Alternaria alternata*. Third and fourth leaves of *Nicotiana benthamiana* plants were toothpick inoculated with *Agrobacterium tumefaciens* carrying the indicated viral constructs. After 11 days, the upper leaves showing systemic viral symptoms were challenged with 10 μl of germination solution (20 mM glucose, 20 mM potassium phosphate) supplemented with 10^7^ spores/ml of *A. alternata*. **(A)** Representative pictures of necrotic lesions (NLS) observed 6 days after *A. alternata* inoculation (DPI) on the indicated PVX-transformed *N. benthamiana* plants. **(B)** Severity of necrotic lesions recorded 4 and 6 days after *A. alternata* infection on the indicated PVX-transformed *N. benthamiana* plants. Values are the means of four independent inoculations repeated on at least three plants; error bars represent the standard deviation. Mock inoculated plants (H_2_O), PVX and PVX::*gfp* transformed plants were used as negative controls. Different letters indicate significant differences (*P* < 0.01) among treatments at each DPI according to One-way ANOVA with *post-hoc* Tukey HSD Test. **(C)** *In vitro* inhibitory activity of total soluble proteins (TSP) extracted from PVX-transformed *N. benthamiana* leaf areas toward *A. alternata*. Fungal growth inhibition was measured as the reduction of the optical density at 550 nm relative to the control treatment (TSP extracted from PVX::*gfp* transformed plants). Values are the means of triplicate determinations; error bars represent the standard deviation. Different letters indicate significant differences (*P* < 0.05) among the different treatments according to One-way ANOVA with *post-hoc* Tukey HSD Test.

To evaluate the effect of Kunitz and Pin1 expression on plant resistance toward fungal pathogens, plants systemically transformed with PVX::*gfp* and PVX::*PIs* were challenge-inoculated with suspensions of *B. cinerea* and *A. alternata* spores. Necrotic symptoms 6 days postinoculation are shown in Figures 2A, 3A. The size of the disease lesions was also measured after 2, 4, and 6 days in the case of *B. cinerea* and after 4 and 6 days in the case of *A. alternata* infection (Figures 2B, 3B).

While *B. cinera* symptoms developed markedly on PVX::*PPI3A2* transformed plants, those expressing the *PKI1* or *PKI2*, *PPI3B2*, and *PPI2C4* genes exhibited partial or almost complete disease resistance, respectively (Figure 2A). Noteworthy, the reduction in the severity of necrotic lesions ranged from 87% (PVX::*PKI2*) to 100% (PVX::*PPI3B2*) 6 days after spore inoculation when compared to the control (PVX::*gfp*) treated plants (Figure 2B).

Differently from *B. cinerea* infection, no symptoms developed on any of the *A. alternata* challenged leaves 2 days post-infection (data not shown). Marked necrotic areas started to appear 4 days post-infection on PVX::*PPI3B2*, PVX::*PPI2C4*, and PVX::*gfp* transformed plants, while those expressing *PKI2*, *PKI1*, and *PPI3A2* genes exhibited a significant increase of disease resistance (PVX::*PKI1*, 84%; PVX::*PKI2*, 93%; PVX::*PPI3A2*, 68%). Six days post-infection all PI expressing plants showed significantly increased resistance (varying between 50 and 66%) when compared to the control ones (PVX::*gfp*) (Figure 3B).

To understand if the inhibition of *B. cinerea* and *A. alternata* growth on PI-expressing *N. benthamiana* plants depended on the chemical composition of leaf TSP, the *in vitro* inhibitory activity of TSP extracted from PIs-transformed *N. benthamiana* leaf areas was compared to that of PVX::*gfp* transformed ones. Data reported in Figures 2C, 3C show that all TSP extracted from PI-transformed plants were able to reduce the growth of both fungal pathogens, except those from *PPI3A2*-expressing plants that selectively inhibited *A. alternata* but not *B. cinerea* proliferation.

### Transient Expression of *PKI2*, *PPI3A2*, and *PPI2C4 PI* Genes Increases Plant Resistance Toward the Bacterial Pathogen *Pseudomonas syringae* pv. *tabaci*

**FIGURE 4:**
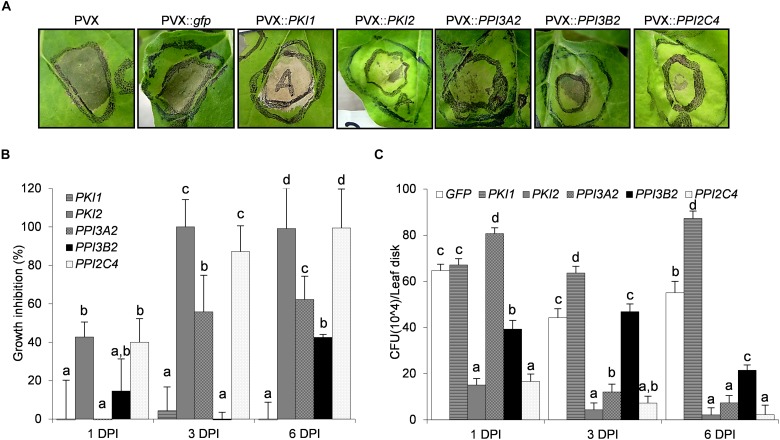
Transient expression of *PKI2* Kunitz inhibitor and of *PPI3A2* and *PPI2C4* Pin1 inhibitor genes increases plant resistance toward the bacterial pathogen *Pseudomonas syringae* pv. *tabaci*. Fully developed leaves of *Nicotiana benthamiana* plants were syringe-infiltrated with a solution of *Agrobacterium tumefaciens* carrying the indicated viral constructs. After 5 days, the previously transformed leaf areas were challenge infiltrated with 40 μl of a 1 × 10^8^ *P. syringae* pv. *tabaci* cells/ml culture **(A)** or needle-pricked and 10 μl of the same *P. syringae* cell suspension were applied at the center of the wounded area **(B,C)**. **(A)** Representative pictures of chlorotic and necrotic symptoms observed 7 days after *P. syringae* pv. *tabaci* infiltration. **(B,C)** The number of surviving bacterias 1, 3, and 6 days after leaf infection with *P. syringae* was determined. Leaf disks (0.8 cm diameter) were excised from the inoculated areas, ground in 10 mM MgSO_4_ and homogenates were either serially diluted, plated and colonies counted on LB-rifampicin agar **(C)** or mixed with the LB-rifampicin and optical density (OD) at 550 nm determined after 16 h of incubation **(B)** (*P* < 0.001). *P. syringae* growth inhibition in **(B)** is expressed in percentage as the reduction in OD of the PVX::*PI* treated samples compared to the control ones (PVX::*gfp*). In all cases values are the means of four independent inoculations repeated on at least three different plants; error bars represent the standard deviation. Mock inoculated plants (H_2_O), PVX and PVX::*gfp* transformed plants were used as negative controls. Different letters indicate significant differences (*P* < 0.01) among the different treatments according to One-way ANOVA with *post hoc* Tukey HSD Test.

To determine whether *Kunitz* and *Pin1* gene overexpression confers protection against bacterial phytopathogens in *N. benthamiana* plants, a cell suspension of *P. syringae* pv. *tabaci* was syringe-infiltrated into the abaxial side of PVX::*PIs* or PVX::*gfp* transformed leaf areas. Chlorotic and necrotic symptoms markedly developed on PVX::*gfp* and PVX::*PKI1* transformed plants 7 days post-infection (Figure 4A). Interestingly, while a mild reduction of symptoms was observed on PVX::*PPI3A2*- and PVX::*PPI3B2*-infected plants, in those expressing the *PKI2* or the *PPI2C4* gene only a small necrosis surrounding the point of inoculation, comparable to that observed in the water inoculated leaves (data not shown), was visible (Figure 4A).

As necrotic lesions showed an irregular outline and it was difficult to exactly determine the area of necrosis, the number of surviving *P. syringae* cells in infected leaves was quantified (Figures 4B,C). Leaf discs were collected from distinct infiltrated areas from each treated plant, homogenized and plated on LB-rifampicin agar at three different dilutions (10^–3^, 10^–4^, and 10^–5^). Alternatively, a 10^–3^ dilution was mixed with LB-rifampicin in 96 well-plates and incubated with constant shaking at 28°C for 16 h. Colony counting and optical density readings retrieved similar results, reported in Figures 4B,C. No inhibition of bacterial growth was observed 6 days after inoculation in PVX::*PKI1*-transformed plants when compared to control ones (PVX::*gfp*). Strikingly, a strong reduction of *P. syringae* population was detected in plants transformed with the PVX::*PPI3B2* and PVX::*PPI3A2* vectors (∼40–60%), and complete resistance was observed in those transformed with the *PKI2* and *PPI2C4* genes. As expected, mock infiltrated leaves showed no necrotic symptoms and no bacterial colonies grew after plating their homogenates.

### Transient Expression of *PPI2C4* and *PKI2* Genes Alters Plant Development

**FIGURE 5:**
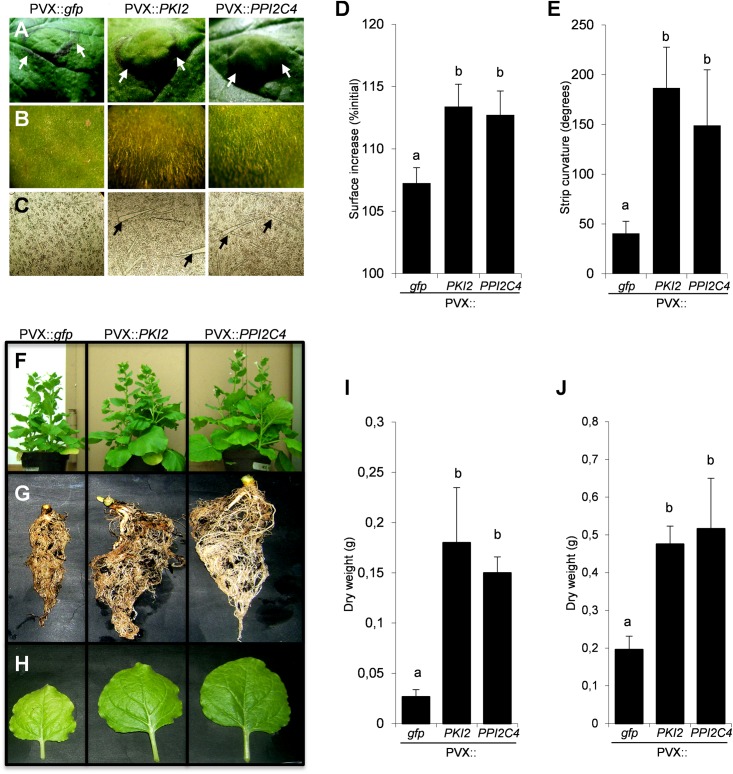
Transient expression of *PKI2* and *PPI2C4* genes enhances plant growth and trichome accumulation. **(A,B)** Representative pictures of *Nicotiana benthamiana* transformed leaf areas 21 days after syringe-infiltration with a solution of *Agrobacterium tumefaciens* carrying the indicated viral constructs. Note the leaf area enlargement **(A)** and overaccumulation of trichomes **(B)** in PVX::*PKI2* and PVX::*PP12C4* transformed leaves. **(C)** Stereomicroscopic view (40X) of adaxial leaf epidermal cells shows higher number and bigger size of trichomes in PVX::*PKI2* and PVX::*PPI2C4* transformed leaf areas. **(D,E)** Curvature of leaf strips and area increase of leaf discs. Leaf strips and discs (1 × 1 cm) from transiently transformed leaf areas (7 days after *A. tumefaciens* syringe-infiltration) were incubated for 24 h as indicated in section “Materials and Methods”. Images were taken before and after incubation and the strip curvature **(E)** and the increase of leaf disc area **(D)** (expressed as a percentage of the initial leaf disc area) were calculated. Values are the means of 15 different leaf strips or discs measurements repeated in three independent experiments; error bars represent the standard deviation. Different letters indicate significant differences (*P* < 0.01) according to One-way ANOVA with *post hoc* Tukey HSD Test. **(F–J)** Effects of systemic *PKI2* and *PPI2C4* over-expression on plant growth. Third and fourth leaves of 2–3-week-old *N. benthamiana* seedlings were tooth-pick incoculated with the indicated viral constructs. PVX or PVX::*gfp* transformed plants were used as controls. Representative pictures of entire plants **(F),** root systems **(G)** and newly formed leaves **(H)**, and measurement of root **(I)** and shoot **(J)** dry weight 21 days after *A. tumefaciens* inoculation. Values are the means of 10 different measurements repeated in three independent experiments; error bars represent the standard deviation. Different letters indicate significant differences (*P* < 0.01) according to One-way ANOVA with *post hoc* Tukey HSD Test.

PVX::*PKI2* and PVX::*PPI2C4* transiently transformed *N. benthamiana* plants showed, in contrast to the untransformed and PVX::*gfp* transformed plants, several developmental abnormalities. Hypertrophy and unusual accumulation of trichomes were observed in locally transformed leaf areas 21 days after syringe-infiltration (Figures 5A–C). Because these macroscopic phenotypes are indicative of a role of PKI2 and PPI2C4 serine PIs in the regulation of cell division and plant development, we tested whether locally transformed *N. benthamiana* leaves showed higher rates of cell expansion. Interestingly, leaf discs from PVX::*PKI2* and PVX::*PPI2C4* transformed plants expanded twice as much as the control ones (PVX::*gfp*) in a 24 h incubation period (Figure 5D). In an additional set of experiments, leaf strip curvature or epinasty, a phenotype often related to cell expansion (Keller and Van Volkenburgh, 1997), was analyzed by measuring the curvature of leaf strips excised from PVX-transformed patches 24 h after incubation. Notably, PPI2C4- and PKI2-expressing leaf strips bent 3–4-fold more than GFP-expressing ones (Figure 5E), indicating leaf asymmetrical expansion, with the adaxial surface growing faster than the abaxial one.

To confirm the relevance of PKI2 and PPI2C4 proteins in the regulation of plant development, *N. benthamiana* seedlings were systemically transformed with the PVX::*PKI2* and PVX::*PPI2C4* viral constructs. Morphological analyses showed that PKI2- and PPI2C4-overexpressing plants grew faster, developed bigger root systems and leaves and exhibited an increase in root and shoot dry weight of more than three and two times, respectively, when compared to controls (Figures 5F–J).

### Effects of PI Expression on *Nicotiana benthamiana* Endogenous Protease Activity, Epidermal Cell Expansion, and Division

**FIGURE 6:**
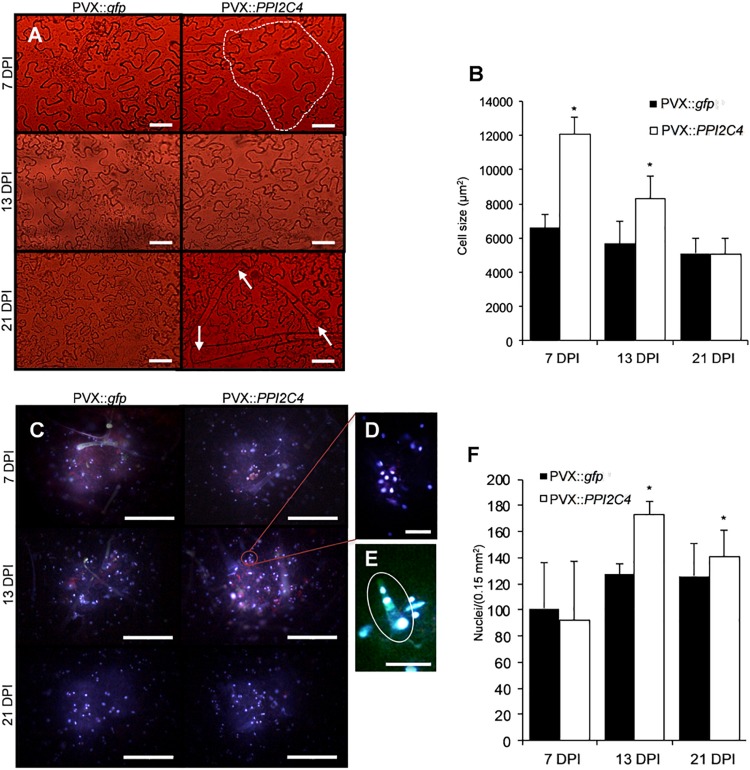
Effect of *PPI2C4* gene overexpression on epidermal cell division and trichome accumulation. **(A,C,D,E)** Representative pictures of *Nicotiana benthamiana* transformed epidermal cells under light microscopy **(A)** or fluorescence microcopy (DAPI staining) **(C–E)**, 7, 13 and 21 days after syringe-infiltration (DPI) with a solution of *Agrobacterium tumefaciens* carring the indicated viral constructs. **(D)** Magnification of **(C)** to show details of clustered nuclei. **(E)** Magnification of a neoforming trichome observed 13 DPI in PVX::*PPI2C4* transformed leaves. Note the bigger size of epidermal cells 7 DPI (circled) and number of trichomes 21 DPI (pointed with arrowheads) in PVX:: *PPI2C4* transformed leaves. Scale bar, 10 μm in **(A,D,E)** and 50 μm in **(C)**. **(B,F)** Cell size **(B)** and number of nuclei **(F)** in transformed adaxial leaf epidemis (*n* = 500 cells, in at least four leaves) 7, 13, and 21 DPI (*P < 0.0001 versus PVX::*gfp* according to Yates’ corrected chi-squared test).

To ask whether the hypertrophic phenotype observed in PKI2 and PPI2C4 expressing leaves reflects induction of cell division, we performed a detailed microscopic analysis of the adaxial epidermis of PVX::*PPI2C4* and PVX::*gfp* agroinfiltrated patches 7, 13, and 21 DPI. While no alterations of cell morphology could be observed, an increase in the average cell size before (7 DPI) and in nuclear density after (13 DPI) was detected in PPI2C4 expressing plants (Figures 6A–C,F). Accordingly, *PPI2C4* expression also lead to the appearance of tight clusters of small-sized cells, some of which being trichomes (Figures 6D,E), 13 DPI. Similar results were obtained in PVX::*PKI2* expressing leaves (data not shown). As expected, none of these changes was observed in control PVX (data not shown) or PVX::*gfp* transformed leaves.

**FIGURE 7:**
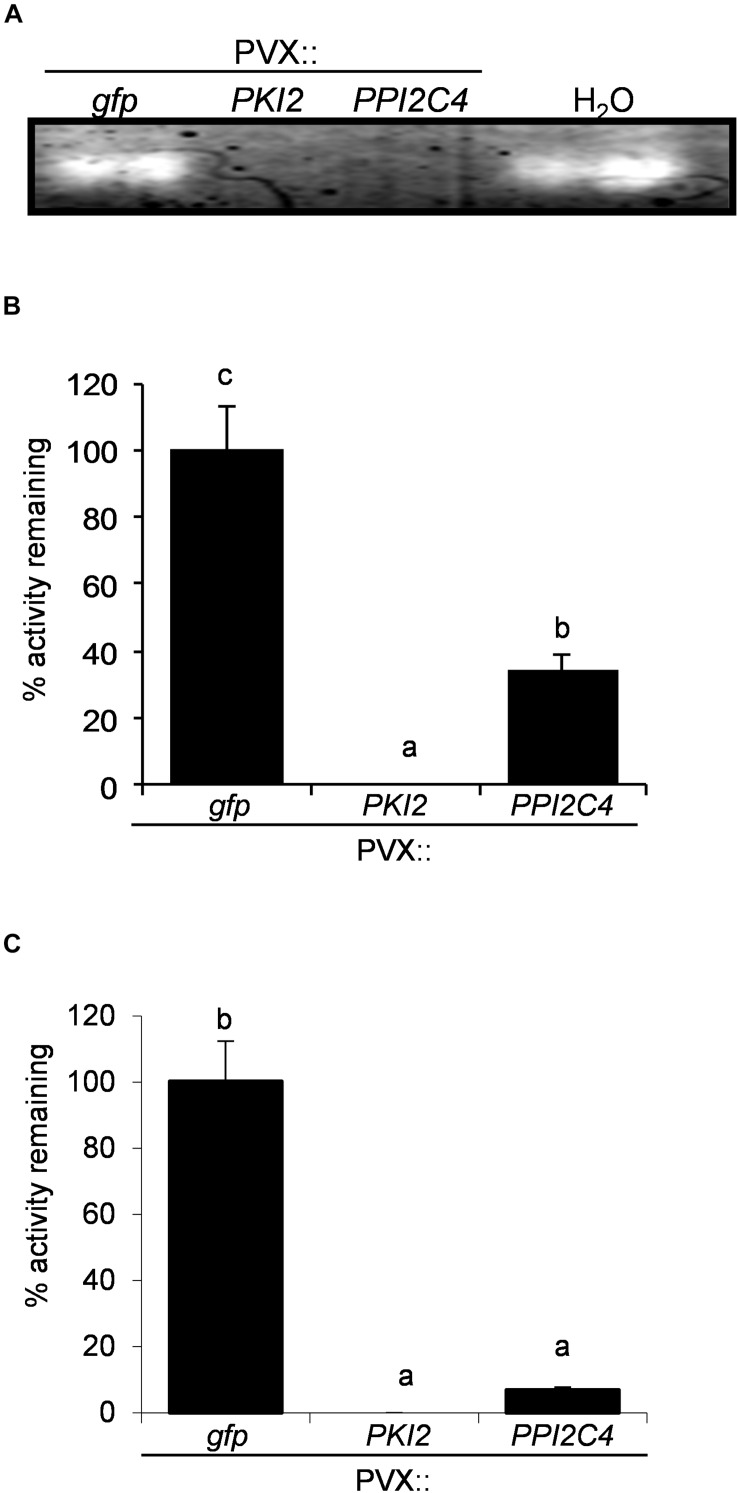
*PKI2* and *PPI2C4* inhibit *Nicotiana benthamiana* apoplastic proteasic activity. **(A)** Zymogen in-gel protease assays of total soluble proteins (TSP) extracted from mock- (H_2_O) and PVX-infiltrated *Nicotiana benthamiana* leaf areas (7 days after syringe-infiltration). **(B,C)** TSP **(B)** or intercellular fluids **(C)** isolated from untransformed *N. benthamiana* plant leaves were incubated with TSP extracted from PVX-transformed *N. benthamiana* leaf areas (7 days after syringe-infiltration) and the remaining protease activity was analyzed by measuring the change in optical density at 450 nm after 1 h of incubation in the presence of a 1% (w/v) azocasein solution. Values are the means of triplicate determinations; error bars represent the standard deviation. Different letters indicate significant differences (*P* < 0.05) according to One-way ANOVA with *post hoc* Tukey HSD Test.

To understand if misregulation of cell division in these plants was associated with an alteration of the endogenous protease activity, TSP from mock- (H_2_O) and PVX-infiltrated *N. benthamiana* leaf areas were either directly used in a in-gel protease assay (Figure 7A) or the first extracts mixed with the latter’s to measure the residual protease activity (Figure 7B). Similar results were obtained in these two assays. In the first case, an additional protease band was clearly visible in the GFP-expressing sample and in the mock-inoculated control, but not in the TSP from PVX::*PKI2* and PVX::*PPI2C4* transformed plants (Figure 7A). In the second case, TSP from PKI2- and PPI2C4-expressing samples showed 100 and 60% higher inhibitory activities, respectively, than those from control plants (GFP expressing). Interestingly, TSP from PKI2- and PPI2C4-expressing samples also showed a high degree of inhibition (90%) toward *N. benthamiana* apoplastic protease activity, when compared to the GFP-expressing controls (Figure 7C).

## Discussion

Hydrolysis and protein synthesis, as well as the regulation of these physiological processes, are fundamental phenomena impacting both plant development and susceptibility/resistance to pathogens (Rawlings et al., 2004, 2014; Turrà and Lorito, 2011; Grosse-Holz and van der Hoorn, 2016). Indeed, several members of the serine protease group, a widely distributed set of extracellular and intracellular proteolytic enzymes, act as pathogenicity factors in different plant pathogens including fungi, oomycetes, bacteria, insects, and nematodes (Ryan, 1990; Urwin et al., 1995; Atkinson et al., 2003; Gvozdeva et al., 2004; Lopez-Solanilla et al., 2004; Hermosa et al., 2006; Pekkarinen et al., 2007; Luo et al., 2009; Thomas and van der Hoorn, 2018). Besides, serine proteases also regulate a panoply of endogenous processes in plants including innate immunity, cell death and nitrogen uptake (Kohli et al., 2012; Grosse-Holz and van der Hoorn, 2016; Salvesen et al., 2016; Balakireva and Zamyatnin, 2018). Interestingly, inhibitors of these enzymes, especially those belonging to the multigene PI families *PKPI* and *Pin1* have been shown to accumulate in plant tissues in a highly precise spatial and temporal manner and following both abiotic and biotic threats (Hermosa et al., 2006; Wang et al., 2008; Singh et al., 2009; Turrà et al., 2009; Boex-Fontvieille et al., 2015; Rustgi et al., 2017). These findings together with the increasing evidence of their inhibitory activity both *in vitro* and *in vivo* toward insects and nematodes, and *in vitro* toward fungi and bacteria is indicative of a possible multitasking activity of these PI families in both the regulation of plant physiology and biochemical defense responses (Duan et al., 1996; Cai et al., 2003; Vila et al., 2005; Turrà and Lorito, 2011; Quilis et al., 2013). However, a clear correlation between *PKPI* and *Pin1* expression and *in planta* modulation of developmental processes or resistance toward fungal or bacterial pathogens is currently missing. We have previously shown that extracellular proteases secreted by the fungal pathogen *B. cinerea* mainly belong to the serine protease class (Hermosa et al., 2006). We further identified, from the complex set of plant-produced PIs, different protein products belonging to the PKPI and Pin1 serine proteinase inhibitor families and showing high inhibitory activity on both fungal growth and disease development when exogenously supplemented to the fungal inoculum source (Hermosa et al., 2006). Now, to demonstrate their efficacy *in vivo* toward fungal plant pathogens, we have heterologously expressed different potato *PKPI* and *Pin1* genes in *N. benthamiana*, a model system to study the effect of transgene expression on plant-pathogen interactions (Goodin et al., 2008). Consistent with our previous findings, all tested *PIs* genes (except the *Pin1 PPI3A2*) efficiently reduced the *in vitro* growth and increased plant resistance toward two fungal pathogens, *B. cinerea* and *A. alternata*. Noteworthy, no *B. cinerea* symptoms developed at all, even 6 days postinoculation, on *N. benthamiana* plants transformed with PPI3B2, the potato Pin1 inhibitor formerly identified for its strong antifungal activity on *B. cinerea in vitro* (Hermosa et al., 2006). To understand if *in planta* over-expression of *PKPI* and *Pin1* genes could also alter plant resistance toward bacterial phytopathogens, we challenged *N. benthamiana* transformed plants with the bacterial pathogen *P. syringae* pv. *tabaci*. Interestingly, bacteria survival in PKI2-, PPI3A2-, PPI3B2-, and PPI2C4-expressing plants dropped over time and necrotic symptoms barely developed on PKI2 and PPI2C4 transformed leaves, suggesting that PKPI and Pin1 PIs might act by exerting either a direct and/or indirect antiproliferative activity on *Pseudomonas*. This hypothesis is supported by two lines of evidence. First, several studies have already shown the importance of *Pseudomonas* spp. cysteine and serine proteases in the degradation of both structural and soluble host plant proteins and virulence (Engel et al., 1998; Axtell et al., 2003; Hotson and Mudgett, 2004). Second, uninfected PKI2-, and PPI2C4-transformed areas from fully developed leaves enlarged abruptly and accumulated higher amounts of trichomes, epidermal cells specialized in defending the plant from both biotic and abiotic stresses (Wagner, 1991; Amme et al., 2005). Importantly, this phenotype was accompanied by an increase in epidermial cell growth and division in PVX:*PPI2C4* agroinfiltrated leaves and an overall shoot and root size in systemically transformed plants, indicative for an endogenous inhibitory activity of these PIs, as also shown by TSP activity on apoplastic proteases.

Overall our findings show for the first time that specific members of the Pin1 and PKPI PI families might act as multifunctional proteins playing fundamental roles in both the regulation of important plant physiological processes such as cell development and differentiation as well as wide-spectrum disease resistance against fungal and bacterial pathogens. These results might represent a framework for the future selection of *Pin1* and *PKPI* genes to be used either individually or in gene pyramiding approaches to obtain fast-growing trees or crops with broad-resistance to biotic threats.

## Data Availability Statement

All datasets generated for this study are included in the article/Supplementary Material.

## Author Contributions

DT, SV, SW, and ML designed and conceived the study, and wrote the manuscript. DT, SV, and RM performed the experiments and analyzed the data. All authors read and approved the final version of the manuscript for publication.

## Conflict of Interest

The authors declare that the research was conducted in the absence of any commercial or financial relationships that could be construed as a potential conflict of interest.

## Footnotes

1 http://www.ncbi.nlm.nih.gov
