# Supplementary material for: Heterologous Expression of PKPI and Pin1 Proteinase Inhibitors Enhances Plant Fitness and Broad-Spectrum Resistance to Biotic Threats
Source: Front Plant Sci. 2020 Apr 30;11:461. doi: 10.3389/fpls.2020.00461 (PMC7204852; doi:10.3389/fpls.2020.00461)
Supplement: Supplementary file 1 [file Data_Sheet_1.docx]

| **Supplementary Table S1.** Primer sets used for RT-PCR and cloning, of the *Kunitz* and *Pin1* genes. | | |
| --- | --- | --- |
| **Primer name** | **Sequence (5' > 3')^1^** | **Corresponding plasmids^2^** |
| CLAPKI | ATCGATTATGAAGTGTTTATTTTTGTT | pGR106::PKI1/pGR106::PKI2 |
| SALPKI | GTCGACTTACTGGACTTGCTTG | pGR106::PKI1/pGR106::PKI3 |
| CLA3B2 | ATCGATTATGGAGTCAAAGTTTGC | pGR106::PPI3A2/pGR106::PPI3B2 |
| SAL3A2 | GTCGACTTAACCAACCACAGGC | pGR106::PPI3A2 |
| SAL3B2 | GTCGACTTAACCGACCACAGGC | pGR106::PPI3B2 |
| CLA2C4 | ATCGATTATGGAGTCAAAGTTTTC | pGR106::PPI2C4 |
| SAL2C4 | GTCGACTTAATTGCTTCGGTAATC | pGR106::PPI2C4 |
| ^1^ Forward primers contain a *ClaI* site (ATCGAT), while reverse primers contain a *Sal*I site (GTCGAC). These enable cloning into the *Cla*I and *Sal*I sites of the pGR106 vector. ^2^Some primers amplified more than one gene because the primer regions were identical or highly similar. | | |
